# Perioperative surgery- and anaesthesia-related risks of laparoscopic Roux-en-Y gastric bypass - a single centre, retrospective data analysis

**DOI:** 10.1186/s12871-018-0654-x

**Published:** 2018-12-13

**Authors:** Anna M. Schürner, Giulia Manzini, Marco Bueter, Erik Schadde, Beatrice Beck-Schimmer, Martin Schläpfer

**Affiliations:** 1 Institute of Anaesthesiology, University Hospital Zurich, Raemistrasse 100, 8091 Zurich, Switzerland; 2 Department of Surgery, University Hospital Zurich, Raemistrasse 100, 8091 Zurich, Switzerland; 3 Department of Surgery, Cantonal Hospital Olten, Baslerstrasse 150, 4600 Olten, Switzerland; 4 Institute of Physiology, University Zurich, Winterthurerstrasse 190, 8057 Zurich, Switzerland; 5 Rush University Medical Center, 1653 W. Congress Pkwy, Chicago, IL 60612-3244 USA; 6 Department of Surgery, Cantonal Hospital Winterthur, Brauerstrasse 15, 8401 Winterthur, Switzerland; 7 Department of Anaesthesiology, University of Illinois at Chicago, 1740 West Taylor Street Suite 3200W, MC 515, Chicago, IL 60612 USA

**Keywords:** Anaesthesia, Bariatric surgery, Postoperative complications, Intraoperative complications

## Abstract

**Background:**

Conservative obesity treatment often leads to limited results. Bariatric surgery is highly efficient, but the candidates are at risk of developing perioperative complications. Bariatric outcomes have been well described in the past, but there are only few reports of perioperative outcomes.

The aim of this study was to evaluate the incidence of anaesthetic and surgical complications of Roux-en-Y bypass.

**Methods:**

Data of all adult patients, who underwent primary Roux-en-Y gastric bypass surgery between 1/2006 and 12/2013 at the University Hospital Zurich were analysed. Using our clinical database, anaesthetic and surgical complications during the first 30 days after surgery were analysed and risk factors determined by multivariate analysis.

**Results:**

Seven hundred eleven patients (72% female, median age 40 years) were analysed. Overall, surgical complications occurred in 34% patient, while complications attributable to anaesthesia occurred in 37%. Post-operative nausea and vomiting (PONV) were observed in 34%, intubation-related complications in 4%, and delayed extubation in 2% of our patients. Within the first 30 days after surgery, 22% of the patients presented with an infection. Gastrointestinal complications were found in 8%, and bleeding complications in 3%. Anaesthesia complications were less common in older patients and in patients anaesthetized with a volatile anaesthetic. Severe complications were more common in patients with a lower body mass index (BMI) and with longer surgery. The risk to develop a postoperative infection was higher in diabetic patients.

**Conclusion:**

Roux-en-Y bariatric surgery has few anaesthetic complications, the most common is PONV. PONV is more common in younger patients and not more common with volatile anaesthetics. Major complications are overall rare and occur in patients with lower BMI and longer surgery, likely reflecting more difficult procedures.

**Electronic supplementary material:**

The online version of this article (10.1186/s12871-018-0654-x) contains supplementary material, which is available to authorized users.

## Background

Obesity is a chronic and multifactorial disease with a disproportional increase in adipose tissue. It is graded into three classes: patients with a body mass index (BMI) of 30.0–34.9 kg/m^2^ are referred to as obese patients class I, BMI 35.0–39.9 kg/m^2^ as class II, BMI > 40 kg/m^2^ as class III [1]. Class III patients may be further subdivided into super obese patients, defined by a BMI of > 45 or > 50 kg/m^2^ [2]. The prevalence of obesity has doubled since 1980 [3], and is posing an increasing challenge to healthcare systems worldwide.

Conservative therapeutic approaches for obese patients have led to limited results in terms of long-term weight loss [4]. In contrast, bariatric surgery is an effective treatment, which may reduce associated diseases such as diabetes mellitus type 2 (T2DM), dyslipidaemia or obstructive sleep apnoea (OSAS) [5–14] and even mortality rates of morbidly obese patients [7, 15].

Among the most frequently performed bariatric procedures is the Roux-en-Y gastric bypass (RYGB) [16]. From a surgical perspective, bariatric surgery in general and RYGB in particular are characterized by a low surgical risk profile and can be performed with low morbidity and low mortality rates [7, 17]. From the perspective of the anaesthesiologist however, only limited data exist regarding the perioperative anaesthesia risk and management of patients with morbid obesity.

The aim of this prospectively collected retrospective cohort analysis is to assess the anaesthesia and surgery-related perioperative risk of obese patients within the first 30 days after RYGB surgery at our university centre between 2006 and 2013.

## Methods

### Data collection

After approval of the local ethics committee in Zurich (Kantonale Ethikkomission Zurich, Switzerland, Chaired by Peter Meier-Abt, KEK-ZH-No. 2015–0260; 14th of July, 2015) we scanned the database of the bariatric surgery in our centre well as our clinical databases (KISIM, Cistec, Zürich) for all patients that underwent a primary laparoscopic RYGB between 01/2006 and 12/2013. Patients with an age below 18 years and those that specifically declared their unwillingness to have their clinical data collected for research purposes were excluded.

For all other patients, patient characteristics (age, gender, BMI, the American Society of Anesthesiologist, ASA, physical status classification) as well as comorbidities (arterial hypertension, T2DM, dyslipidaemia, obstructive sleep apnoea syndrome, OSAS, chronic obstructive pulmonary disease, COPD, and reflux/hiatal hernia) and pre-existing medication were evaluated.

Surgery specific data were also analysed (surgery time, the conversion rate to open surgery, and blood loss).

The anaesthesia specific characteristic’s included: anaesthesia duration, anaesthesia monitoring (arterial/ central venous and pulmonary venous catheters), anaesthesia medication (opioid, hypnotic agent, muscle relaxant, catecholamines, and antihypertensives), method of intubation (conventional laryngoscopy vs facilitated method with SensaScope® or fiberoptic) and airway anatomy (Mallampati and Cormack&Lehane classes), volume replacement (crystalloid, colloid, packed red blood cells) and diuresis.

The following anaesthesia-related complications were collected: intubation complications (unexpected difficult airway, secondary change of intubation technique, failed intubation), the need for re-intubation or delayed extubation, complications related to catheter insertion and positioning during surgery; medication-related adverse reactions as well as the prevalence of postoperative nausea and vomiting (PONV).

Intra- and postoperative surgical complications, as well as complications within the first 30 days after the intervention were registered according to the Clavien-Dindo Classification [18]. The complications were classified by type into gastrointestinal complications (e.g. anastomotic stenosis or leak, incisional hernia, fluid collection and anastomotic ulcer), infections (wound infections, general infections, urinary tract infections, pneumonia), bleeding complications, cardiovascular (tachycardia, myocardial infarction), respiratory (pleural effusion), and renal complications were monitored.

We also recorded the length of hospital stay, the number of days in the intensive care unit (ICU) and the numbers of hospital re-admissions during the first 30 postoperative days.

### RYGB surgery

All RYGB operations were performed laparoscopically in a standardised way with the circular stapler technique for the gastro-enteral anastomosis as previously described [19].

### Statistical analyses

Statistic calculations were performed with GraphPad Prism 6.0 for Mac (GraphPad Inc., La Jolla, CA), Microsoft Excel for Mac, Version 15.40 (Microsoft Corporation, Redmont, WA), and JMP 10.0.2 by SAS Institute, Cary, N.C., USA. Data are presented as median and interquartile range (IQR) for continuous nonparametric data, and mean and standard deviation for parametric data. Kolmogorov-Smirnov test was used to test for distribution. Categorical data were presented in percentages. Missing data points are disclosed in the tables.

Outcomes of interest were identified and an univariate analysis was performed with dichotomous covariates using logistic regression. C-statistic was performed and the Youden index was used to dichotomize continuous covariates. To correct for potential confounders, a multivariable model was constructed. Covariates with a significant correlation in the univariate analyses (*p* < 0.1), as well as covariates of clinical interest were included in the model. A *p*-value < 0.05 was considered statistically significant.

## Results

In total, 712 patients underwent a primary standard laparoscopic RYGB between 01/2006 and 12/2013. One single patient had refused data collection for research purposes and was thus excluded.

### Patient and procedure characteristics

The median age was 40 (32–49) years with 72% female and 28% male patients and a median BMI of 45 (41–49) kg/m^2^. The patient’s anaesthesia-related risk was estimated using the ASA classification. Four hundred and sixteen patients (60%) patients were classified as ASA II, 278 (40%) as ASA III, 5 patients (1%) as ASA IV. Baseline characteristics of patients, intraoperative surgery and anesthesia parameters are summarized in Table 1.

**Table 1:** Patient characteristics and intraoperative parameters related to surgery and anaesthesia

Full cohort *n* = 711		Missing data
Age (yrs.), median (IQR)	40 (32–49)	12
Sex, n (%)		12
Male	199 (28)	
Female	500 (72)	
BMI (kg/m^2^), median (IQR)	45 (41–49)	12
ASA, n (%)		12
II	416 (60)	
III	278 (40)	
IV	5 (1)	
Comorbidities, n (%)		12
Hypertension	353 (50)	
T2DM	200 (29)	
Dyslipidaemia	89 (13)	
OSAS	144 (21)	
COPD	32 (5)	
Reflux/hiatal hernia	352 (50)	
Previous gastric banding, n (%)	83 (12)	12
Surgery time (min), median (IQR)	145 (120–173)	12
Gastric bypass, n (%)		25
Laparoscopic	676 (99)	
Conversion to open	10 (1)	
Blood loss (ml), median (IQR)	25 (10–50)	14
Monitoring, n (%)		12
Arterial Catheter	540 (77)	
Central venous catheter	98 (14)	
Pulmonary artery catheter	3 (< 1)	
Intubation, n (%)		16
Rapid sequence induction, conventional laryngoscopy	360 (52)	
Awake fibreoptic	215 (31)	
Conventional induction, conventional laryngoscopy	69 (10)	
Rapid sequence induction with Sensascope®	28 (4)	
Conventional induction, with Sensascope®	13 (2)	
Conventional induction, asleep fibreoptic	9 (1)	
Mallampati, n (%)		228
I	146 (30)	
II	216 (45)	
III	100 (21)	
IV	21 (4)	

The median duration of surgery was 145 (120–173) minutes with a necessity for conversion to open surgery in 10 (1%) patients and an estimated blood loss of 25 (10–50) ml.

The anaesthesia duration was 270 (235–310) minutes with desflurane being the predominantly used anaesthetic for maintenance of anaesthesia in 89% of the patients. In 1% of the patients, sevoflurane and in 10% propofol was used. The type of general anaesthetic was determined by the responsible staff anaesthesiologist. The most frequently used intubation method was a rapid sequence induction and conventional laryngoscopy in 52% of the cases, followed by fibreoptic awake intubation in 31%. Conventional induction with conventional laryngoscopy was performed in 10%, in 6% with the Sensascope® and in 1% as fibreoptic intubation.

Details about the patient’s co-medication and about co-administered intraoperative drugs can be found in Additional files 1 and 2.

### Anaesthesia-related complications

The percentage of patients experiencing anaesthesia-related complications was 37%. The most prominent adverse event was PONV with 34%, followed by intubation/extubation-related complications (6%). Of note is that some patients suffered from several complications, which explains why the sum of the different subgroups exceeds the overall number of patients with complications.

#### Intubation-related events

4% of the patients experienced a complication related to the intubation period. One (< 1%) patient had an aspiration, and 1 (< 1%) patient suffered from a bronchospasm. Three (< 1%) patients presented with a desaturation and another 3 (< 1%) had epistaxis due to the intubation manoeuvre, in 1 (< 1%) patient the tube was misplaced into the main bronchus and had to be withdrawn. An alternative (compared to the planned) intubation procedure had to be established in 2% of the patients: in 6 (1%) patients a fibreoptic procedure was established instead of the conventional procedure, in 3 (< 1%) patients a conventional intubation was favoured over the planned fibreoptic method, and 2 (< 1%) patients in whom a conventional intubation was unsuccessful, were allowed to wake up, followed by an awake fibreoptic intubation in a second approach.

#### Extubation-related events

Postoperative re-intubation was necessary in 3 (< 1%) patients due to pulmonary decompensation in 2 patients and bleeding from the surgical anastomosis in 1 patient. Two percent of the patients were not extubated immediately after surgery, but on the ICU. The reason for the prolonged weaning were difficult intubation in 1% of the patients, difficulties to maintain an appropriate oxygenation in 3 (< 1%) patients, unplanned conversion to open surgery in 3 (< 1%) patients, over sedation in 1 (< 1%) patient and planned delayed extubation because of pre-existing medical conditions in 4 (1%) patients. Details of patients with anaesthesia complications are given in Additional file 3.

#### Airway problems and high BMI

The incidence of an in- and/ or extubation related event was 6% in both groups: the super obese patients (BMI > 45 kg/m^2^) and patients with a BMI ≤ 45 kg/m^2^ (*p* = 0.9).

#### Adverse drug reactions

There were 4 (< 1%) patients with an adverse drug reaction: 2 patients experienced a hypotension and rash due to histamine liberation after atracurium use, 1 patient presented with hypotension after infusion of metamizole and 1 patient developed bronchospasm after infusion of glycopyrronium-neostigmin for muscle relaxant reversal. Even though 50% of the patients were administered PONV prophylaxis, 34% of the patients suffering from PONV.

#### Evaluation of predictors of anaesthesia complications

We evaluated the impact of patient age, duration of surgery, BMI, ASA classification status, the type of anaesthesia, and catecholamine and PONV prophylaxis on the occurrence of anaesthesia complications.

Patients older than 35 (*p* < 0.001) and patients with volatile anaesthetics (*p* < 0.001) had a lower likelihood to experience anaesthetic complications at all, Table 2.

**Table 2:** Potential predictors of overall anaesthesia complications (including PONV)

Covariate	OR and CI	*p*-value
Age (>35y)	0.55 (0.38–0.76)	< 0.001*
Surgery time (> 130 min)	1.26 (0.91–1.75)	0.2
BMI (> 44 kg/m^2^)	0.78 (0.55–1.07)	0.1
T2DM	1.02 (0.71–1.47)	0.9
Anaesthesia (volatile vs. i.v.)	0.31 (0.18–0.51)	< 0.001*
Catecholamine use	1.01 80.67–1.52)	1.0

* indicates significant factors with a *p* < 0.05

Patients older than 35 years had a lower incidence of PONV (43 vs 29% OR 0.53, *p* < 0.001), PONV was more frequent in females (38 vs 23%, OR 1.91, *p* < 0.001), and less frequent when volatile anaesthesia was used (OR 0.31, *p* < 0.001), Table 3.

**Table 3:** Potential predictors of PONV

Covariate	OR and CI	*p*-value
Age (> 35 years)	0.53 (0.37–0.76)	< 0.001*
Gender (f vs. m)	1.91 (1.30–2.87)	< 0.001*
Surgery time (> 130 min)	1.28 (0.91–1.82)	0.15
BMI (> 44 kg/m2)	0.73 (0.52–1.02)	0.07
Anaesthesia (volatile vs. i.v.)	0.33 (0.20–0.56)	< 0.001*
T2DM	1.00 (0.67–1.46)	1.0
Application of antiemetic drug	1.12 (0.80–1.57)	0.50

* indicates significant factors with a *p* < 0.05

### Surgical complications in the first 30 postoperative days

The number and the severity of complications according to the Clavien-Dindo scoring system [18] (within the first 30 postoperative days) is given in Table 4.

**Table 4:** Patient’s highest complication according to Clavien-Dindo classification within the first 30 days after surgery

Highest complication per patient in the first 30 days according to Clavien-Dindo classification Full cohort, n = 711	n (%)	Missing data (n)
None	452 (66)	22
With complication	237 (34)	22
I	36 (5)	22
II	113 (16)	22
IIIa	41 (6)	22
IIIb	47 (7)	22
IVa	0 (0)	22
IVb	0 (0)	22
V	0 (0)	22

No surgical complication was observed in 66% of the patients. Of the remaining patients, 5% had a complication grade I, 16% grade II, 6% grade IIIa and 7% grade IIIb. There were no patients with grade IVa or IVb complications, and no mortality (complication grade V).

Patients with Clavien-Dindo complications grade IIIb had to be surgically reexplored for the following reasons: infections in 4% (*n* = 27), anastomotic leak in 1% (*n* = 8), haematoma evacuation or active bleeding in 1% (*n* = 7), incisional hernia in 1% (*n* = 4). One patient had to undergo a tracheostomy because of respiratory insufficiency.

Detailed information about the type and number of surgical complications within the first 30 days after surgery is given in Additional file 4. We observed gastrointestinal, infectious, bleeding, and cardiovascular complications in 8,22, 3, and 1% of patients respectively. Respiratory and renal problems occurred in < 1% of the patients and other problems occurred in 2% of the patients (pain, exanthema). It is important to note, that patients who developed more than one complication were counted multiple times.

#### Evaluation of predictors of surgical complications

The impact of age, duration of surgery, BMI, T2DM, the type of anaesthesia, and the use of catecholamines on the occurrence of major surgical complications (defined according Clavien-Dindo grade IIIb and higher) was examined. Multivariate analysis showed, that a lower BMI was associated with higher incidence of major surgical complications (15 vs 6%, OR 2.46, *p* = 0.04) and that a procedure lasting longer than 170 min was associated with more complications (12 vs. 5%, OR 0.44, *p* = 0.01), Table 5.

**Table 5:** Potential predictors of major surgical complications

Covariate	OR and CI	*p*-value
Age (>42y)	0.85 (0.45–1.63)	0.6
Surgery time (> 170 min)	2.27 (1.20–4.23)	0.01*
BMI (> 37 kg/m^2^)	0.46 (0.19–0.97)	0.04*
T2DM	1.42 (0.71–2.75)	0.3
Anaesthesia (volatile vs. i.v.)	1.01 (0.43–2.83)	1.0
Catecholamine use	0.74 (0.36–1.64)	0.4

* indicates significant factors with a *p* < 0.05

As far as infectious complications are concerned, only T2DM was associated with a higher incidence (20 vs. 29%, OR 1.60, *p* = 0.02), Table 6.

**Table 6:** Potential predictors of infections

Covariate	OR and CI	*p*-value
Age (>41y)	1.24 (0.84–1.82)	0.3
Gender (f vs. m)	1.50 (0.99–2.32)	0.05
Surgery time (> 165 min)	1.45 (0.98–2.13)	0.06
BMI (> 48 kg/m^2^)	1.29 (0.86–1.91)	0.2
T2DM	1.60 (1.06–2.40)	0.02*

* indicates significant factors with a *p* < 0.05

### Hospitalisation and outcome characteristics

The median hospital stay was 7 (6–8) days for patients after RYGB. The immediate postoperative transfer of the patients to an ICU was necessary in 5% of the cases. In all other cases patients could be extubated in the operating room (OR) and were monitored for a few hours or overnight in the post anaesthesia care unit (PACU), the intermediate care unit (IMC) or to the ICU, according to availability of space. A second surgical intervention within 30 days was necessary in 7% of the patients.

We found 37% patients with a complication related to anaesthesia, in this group approximately one third (12% of the patients) suffered from both: a complication related to anaesthesia and one related to surgery.

## Discussion

In this retrospective cohort study, we found that approximately one third of patients undergoing RYGB surgery in a Swiss university hospital suffer from either an anaesthesia- or surgery-related perioperative complications. The vast majority of these complications are minor and there was no mortality.

### Anaesthesia-related complications

In contrast to peri- and postoperative surgery-related morbidity after RYGB, for which a vast body of evidence can be found in the literature, little is known about anaesthesia-related complications. Only results from smaller patient cohorts [20, 21] are available. The existing studies report an anaesthesia complication rate of 4% [20, 21]. In this prospective database, 4% of the patients experienced intubation-related complications, while a previous study reported only 1% [21]. This difference may be due to differences in measurement. In this study, for example the necessity for intermittent ventilation prior to intubation due to a drop in oxygen saturation was recorded as an event. In analogy to the Clavien-Dindo classification, international consensus definitions of anaesthesia complications are urgently needed to be able to compare data of different studies.

With regard to extubation complications, the results reported are in line with previous reports. Delayed extubation occurred in 2% of the patients, other studies reported 3–4% [20]. In this study, PONV accounted for the highest number of anaesthesia-related complications. As there are no other data available about the incidence of PONV in RYGB, no comparison with the literature was possible. Risk factors for PONV [22] are use of a volatile anaesthetic, women and a young patient age. Interestingly, we found a lower incidence of PONV in patients anaesthetised with a volatile anaesthetic. As desflurane is our standard anaesthetic for RYGB surgery, only patients with a history of PONV have received propofol anaesthesia and this might be the result of a selection bias. The database did not contain information about previous anaesthetic complications to put this factor into the multivariate model. The use of an intraoperative antiemetic did not reduce PONV incidence in the multivariate analysis. Of note, any antiemetic or prokinetic agent administered throughout the course of anaesthesia was registered as antiemetic therapy (any single or combined administration of metoclopramide, mephamesone, droperidol, tropisetron, or ondansetron). Only a systematic and effective PONV regimen implemented at our institution has decreased PONV rates from 51% in the first compared to 15% in the last year of the observation period, which demonstrates the effectiveness of this action (PONV rates over time and details about PONV regimen are given in Additional file 5).

### Surgery-related complications

Although at first sight the overall incidence of 34% of all patients experiencing a complication related to surgery appears high, the rate of specific complications in our patient population is similar or even lower compared to other studies. Stenosis of the gastrojejunostomy has been previously reported to be as high as 9–25%, depending on the technique, that has been used to facilitate the gastrojejunostomy (circular vs. linear technique) [23]. Here, 3% of the patients experienced a stenosis of the gastrojejunostomy in our population. We found a comparable incidence of leaks of the gastrojejunostomy in 1% of patients [24]. Wound infections were observed in 13% of the patients in our centre which is comparable to 9% documented in another study [25].

Of note, no patient died in this cohort (Clavien-Dindo Complication V) while the mortality rate was previously reported to be 0.18% [24] in a metanalysis and systematic review. This is an important achievement of this program, given that the retrospective data analysis includes a learning curve starting in 2006 when the first cases of RYGB were performed in our centre.

Potential risk-factors for developing major surgical complications were evaluated. Interestingly, a lower BMI resulted in a higher rate of major surgical complications, which remains unexplained, but might be a training phenomenon. A longer duration of the procedure resulted in more complications. Which is likely a surrogate marker for a technically more challenging procedure or procedures performed by less experienced surgeons.

The most common type of complications were infections, significantly more common in patients suffering from T2DM. This is in accordance with other studies reporting wound infections as the most common complication and T2DM as an independent risk factor for surgical site infection [26].

This study has several limitations. It is a single centre retrospective analysis. There is an era bias since the analysis spans over 10 years. Surgical complications are collected prospectively at our institution: this results in a complete, yet sensitive database. Complications are discussed weekly at the morbidity and mortality conference, which helps to uncover treatment problems and to implement new treatment strategies. Nevertheless, anaesthetic complications after RYGB have not been reported in much detail before and a large population has been studied.

## Conclusion

In conclusion, our findings suggest that bariatric surgery is performed with low surgery- and anaesthesia-related complication rates. As PONV is our most common anaesthesia complication, we conclude, that the factors affecting PONV had a strong impact on the overall predictors of anaesthesia complication. PONV was more common in younger and female patients. The higher incidence of PONV in the intravenous group is most likely a result of a selection bias.

The majority of surgical complications were low grade (IIIA or less). A higher incidence of severe complications was observed in patients with a longer surgery and with a lower BMI. Patients with T2DM have a higher risk of infectious complications.

## Additional files

Additional file 1: Co-medication and incidence of smoking. (DOCX 14 kb)

Additional file 2: Intraoperative drug administration. (DOCX 15 kb)

Additional file 3: Details of patients with anesthesia complications. (DOCX 14 kb)

Additional file 4: Details of patients with surgical complications. (DOCX 15 kb)

Additional file 5: PONV risk evaluation and treatment details *and* PONV after implementation of a treatment algorithm. (DOCX 16 kb)

## Abbreviations

ASA: American Society of Anesthesiologists physical status classification
BMI: Body mass index
CI: Confidence interval
COPD: Chronic obstructive pulmonary disease
ICU: Intensive Care Unit
IMC: Intermediate Care
IQR: Interquartile range
OR: Odds ratio
OSAS: Obstructive sleep apnoea syndrome
PACU: Post Anaesthesia Care unit
PONV: Postoperative Nausea and Vomiting
RYGB: Laparoscopic Roux-en-Y-Gastric Bypass
T2DM: Type II diabetes mellitus
