# Supplementary material for: Perioperative surgery- and anaesthesia-related risks of laparoscopic Roux-en-Y gastric bypass - a single centre, retrospective data analysis
Source: BMC Anesthesiol. 2018 Dec 13;18:190. doi: 10.1186/s12871-018-0654-x (PMC6293573; doi:10.1186/s12871-018-0654-x)
Supplement: Supplementary file 1 — Co-medication and incidence of smoking. (DOCX 14 kb) [file 12871_2018_654_MOESM1_ESM.docx]

**Additional file 1**

**Co-medication and incidence of Smoking**

| **Full cohort n=711** |  | Missing data |
| --- | --- | --- |
| **Medication, n (%)**  No medication  Beta-blocker  ACE-inhibitor/AT-2 blocker  Diuretic  Statin  Proton pump inhibitor  Oral antidiabetic  Insulin  Antidepressant | 214 (31)  69 (10)  233 (33)  36 (5)  68 (10)  192 (28)  99 (14)  48 (7)  117 (17) | 12 |
| **Smoking, n (%)** | 279 (40) | 17 |
