# Supplementary material for: Perioperative surgery- and anaesthesia-related risks of laparoscopic Roux-en-Y gastric bypass - a single centre, retrospective data analysis
Source: BMC Anesthesiol. 2018 Dec 13;18:190. doi: 10.1186/s12871-018-0654-x (PMC6293573; doi:10.1186/s12871-018-0654-x)
Supplement: Supplementary file 2 — Intraoperative drug administration. (DOCX 15 kb) [file 12871_2018_654_MOESM2_ESM.docx]

**Additional file 2**

**Intraoperative drug administration**

| **Full cohort, n=711** |  | Missing data n (%) |
| --- | --- | --- |
| **Anaesthetic, n (%)**  Desflurane  Propofol  Sevoflurane | 620 (89)  73 (10)  5 (1) | 12 |
| **Muscle relaxant, n (%)**  Rocuronium  Atracurium  Succinylcholine | 381 (55)  372 (53)  160 (23) | 12 |
| **Muscle relaxant reversal, n (%)**  Glycopyronium/neostigmin  Sugammadex | 73 (10)  10 (1) | 12 |
| **Opiates (mg), median (IQR)**  Remifentanyl  Fentanyl | 2.0 (1-2)  1.0 (0.5-1) | 12 |
| **Catecholamines, n (%)**  Ephedrine  Norepinephrine  Neosynephrine  Epinephrine  Dobutamine | 511 (73)  269 (38)  11 (2)  2 (<1)  0 (0) | 12 |
| **Antihypertensives, n (%)**  Clonidine  Nitroglycerin  Urapidil  Beta-blocker | 108 (15)  44 (6)  17 (2)  15 (2) | 12 |
