# Supplementary material for: Perioperative surgery- and anaesthesia-related risks of laparoscopic Roux-en-Y gastric bypass - a single centre, retrospective data analysis
Source: BMC Anesthesiol. 2018 Dec 13;18:190. doi: 10.1186/s12871-018-0654-x (PMC6293573; doi:10.1186/s12871-018-0654-x)
Supplement: Supplementary file 3 — Details of patients with anesthesia complications. (DOCX 14 kb) [file 12871_2018_654_MOESM3_ESM.docx]

**Additional file 3**

**Details of patients with anaesthesia complications**

| **Patients with anaesthesia-related complications**  Full cohort, n=711 | n (%) | Missing data, n |
| --- | --- | --- |
| No complication  With complication | 443 (63)  256 (37) | 12  12 |
| Related to intubation  Delayed extubation  Reintubation  Related to catheter insertion  Postoperative nausea and vomiting  Positional damage  Adverse drug reaction | 28 (4)  16 (2)  3 (<1)  6 (1)  234 (34)  0 (0)  4 (1) | 12  12  12  12  12  12  12 |
