# Supplementary material for: Perioperative surgery- and anaesthesia-related risks of laparoscopic Roux-en-Y gastric bypass - a single centre, retrospective data analysis
Source: BMC Anesthesiol. 2018 Dec 13;18:190. doi: 10.1186/s12871-018-0654-x (PMC6293573; doi:10.1186/s12871-018-0654-x)
Supplement: Supplementary file 4 — Details of patients with surgical complications. (DOCX 15 kb) [file 12871_2018_654_MOESM4_ESM.docx]

**Additional file 4**

**Details of patients with surgical complications**

| **Type of complication** | **n (%)** | Missing data, n |
| --- | --- | --- |
| **Gastrointestinal**  Anastomotic stenosis  Anastomotic leak  Incisional Hernia  Fluid collection (abdominal or seroma)  Anastomotic ulcer  Diarrhoea  Ileus  Ulcer (unspecified)  Foreign body  Gastric perforation  Liver laceration  Nausea  Obstipation  Splenic injury | **59 (8)**  22 (3)  10 (1)  6 (1)  6 (1)  3 (<1)  2 (<1)  2 (<1)  2 (<1)  1 (<1)  1 (<1)  1 (<1)  1 (<1)  1 (<1)  1 (<1) | 22 |
| **Infections**  Wound infections  Infections (general or non-specified)  Urinary tract infection  Pneumonia  Leucocytosis  Tinea corporis | **157 (22)**  94 (13)  40 (6)  15 (2)  6 (1)  1 (<1)  1 (<1) | 22 |
| **Bleeding complications**  Bleeding or drop in haematocrit  Hematoma  Hematemesis | **24 (3)**  16 (2)  7 (1)  1 (<1) | 22 |
| **Cardiovascular**  Tachycardia (including atrial fibrillation)  Hypertension  Myocardial infarction | **8 (1)**  5 (1)  2 (<1)  1 (<1) | 22 |
| **Respiratory**  Pleural effusion  Supraglottic oedema | **3** **(<1)**  2 (<1)  1 (<1) | 22 |
| **Renal**  Renal failure | **2** **(<1)**  2 (<1) | 22 |
| **Others**  Pain (not specified)  Exanthema | **17 (2)**  12 (2)  5 (1) | 22 |
