# Supplementary material for: Perioperative surgery- and anaesthesia-related risks of laparoscopic Roux-en-Y gastric bypass - a single centre, retrospective data analysis
Source: BMC Anesthesiol. 2018 Dec 13;18:190. doi: 10.1186/s12871-018-0654-x (PMC6293573; doi:10.1186/s12871-018-0654-x)
Supplement: Supplementary file 5 — PONV risk evaluation and treatment details and PONV after implementation of a treatment algorithm. (DOCX 16 kb) [file 12871_2018_654_MOESM5_ESM.docx]

**Additional file 5**

**PONV risk evaluation and treatment details**

Risk factors:

1. female gender
2. non-smoker
3. motion sickness or history of PONV
4. necessity of high opioid doses postoperatively

For each risk factor 1 point is assigned

1-2 points: no measures necessary

2 points: intravenous anaesthesia and 4mg dexamethasone at the time of anaesthesia induction; in case of volatile anaesthesia additionally 4mg ondansetron before emergence

3-4 points: intravenous anaesthesia, 4mg dexamethasone at the time of anaesthesia induction, 4mg ondansetron before emergence, in case of volatile anaesthesia additionally 0.5mg droperidol before emergence

**Development of PONV after implementation of a PONV treatment algorithm**

| Year | 2006 | 2007 | 2008 | 2009 | 2010 | 2011 | 2012 | 2013 |
| --- | --- | --- | --- | --- | --- | --- | --- | --- |
| PONV-Incidence [%] | 51.39% | 46.99% | 52.56% | 44.05% | 35.90% | 22.61% | 14.00% | 14.77% |
